# Evaluating the Cost-Effectiveness of Chlorhexidine-Coated vs. Standard Peripheral Insertion Central Catheters in Patients with Hematologic Disease: A Health Economic Analysis

**DOI:** 10.3390/ijerph22030373

**Published:** 2025-03-04

**Authors:** Jia Xu, Hossein Zare, Herng-Chia Chiu, Renan C. Castillo

**Affiliations:** 1 Department of Health Policy and Management, Johns Hopkins Bloomberg School of Public Health, Baltimore, MD 21202, USA; hzare1@jhu.edu (H.Z.); rcastil1@jhu.edu (R.C.C.); 2 Institute for Hospital Management, Tsinghua Shenzhen International Graduate School, Shenzhen 518055, China; chiuhc@sz.tsinghua.edu.cn

**Keywords:** cost-effectiveness analysis, chlorhexidine-coated PICC, catheter-related complication, ICER, one-way sensitivity analyses

## Abstract

Background/Objectives: This study was conducted to assess the cost-effectiveness of chlorhexidine-coated (AGBA) peripheral insertion central catheters (PICCs) versus standard PICCs for managing catheter-related complications among patients with hematologic disease. Methods: A decision tree health economic model was developed, incorporating quality-adjusted life years (QALYs) derived from the literature, as well as complication rates and per-patient costs from a randomized controlled trial. The base case incremental cost-effectiveness ratio (ICER) was assessed against established willingness to pay (WTP) thresholds. One-way sensitivity analyses were conducted to address assumptions and uncertainties. Results: The mean healthcare cost per patient of standard PICCs was RMB 21,987.32 (USD 3242.82, at an average exchange rate of RMB 678.03 = USD 100), affecting 0.68 QALYs in 90 days. The mean healthcare cost per patient of AGBA PICCs was RMB 19,696.23 (USD 2904.92), affecting 0.73 QALYs in 90 days, thus resulting in a saving of RMB 2291.10 (USD 428.44). After the model simulation, standard PICCs resulted in a reduction of 0.05 QALYs. The ICER for AGBA PICCs compared with standard PICCs was consistently centered at RMB 4271.31 (USD 629.96). Conclusions: one-way sensitivity analyses of cost-effectiveness versus WTP confirmed the robustness of the model across various parameter changes, indicating that AGBA PICCs could provide significant healthcare savings over a 1-year period when adopted in routine chemotherapy treatment for patients with hematologic disease.

## 1. Background

Peripherally inserted central catheters (PICCs) are essential tools in modern healthcare and are primarily used to administer chemotherapy or antibiotics. Due to their lower infection rates and longer indwelling times, PICCs are crucial for reducing healthcare-associated infections [1,2]. Different types of PICCs appear to reduce catheter-related complications, with costs increasing relatively. To make the best healthcare decisions, it is important for payers to understand the cost-effectiveness of different PICCs. According to industry data, in 2020, over 2.7 million PICCs were used in the US, while almost 1 million were used in China; this rate has since continued to increase by >20%.

Along with their wide usage, common concerns regarding PICCs include central-line-associated bloodstream infection (CLABSI) and venous thromboembolism [3]. One study investigated the relationship between the indwelling time of PICCs and the CLABSI rate; the median indwelling time was 26 days (range of 0–385), and the CLABSI incidence was 4.0/1000 catheter days [4].

This study focused on two types of PICCs—standard and chlorhexidine-coated (AGBA) PICCs—to compare their efficacy in managing catheter-related complications. Conducted within the hematology department of a Class 3A hospital in China, this health economic model used data from a randomized controlled trial. Several studies from within this hospital reported that the average 4-year overall survival rate was >75% in AML patients who underwent human leukocyte antigen-matched sibling donor transplantation (MSDT) [5,6]. This means that patients require several PICCs during their treatment period, and some severe catheter-related events, such as CLABSI, can impact survival. Common adverse events associated with catheters include catheter occlusion, exit-site infection, venous thromboembolism, and CLABSI [1]. While some adverse events, such as occlusion and suspected infections, may be resolved with appropriate interventions, CLABSI is a significant concern because it requires laboratory-confirmed testing for diagnosis. However, in real-world settings, empiric therapies, including catheter removal and antibiotic administration, are often initiated [7,8].

The standard catheter care protocol involves obtaining blood cultures to test for CLABSI and performing routine catheter maintenance. If the catheter’s function cannot be restored, the catheter must be removed, and a new catheter is typically inserted after a few days. This process can increase costs and cause patient discomfort, highlighting the importance of reducing catheter-related complications [9,10].

The clinical results of chlorhexidine-coated versus standard PICCs showed that 113 patients accepted chlorhexidine-coated PICCs (AGBA PICCs), while 111 accepted standard PICCs. In the AGBA group, one patient required a second puncture, compared to three patients in the standard group; however, this difference was not statistically significant (*p* = 0.304). Regarding CLABSI, unknown fever, and local complications, eight patients met the criteria for BSI, with three of those fulfilling the criteria for CLABSI, and the remaining five were diagnosed with MBI-LCBI. A total of 36 patients exhibited CLABSI-related symptoms, but laboratory test results indicated unknown fever. All three CLABSI cases occurred in the standard PICC group; nonetheless, there was no statistically significant difference between the two groups (*p* = 0.076). In terms of performance concerning unknown fever and local complications, the AGBA PICC group also outperformed the standard group, although this difference was not significant.

During the 90-day treatment, the setting of patients combines hospital and home-based care. Therefore, more accurate and cost-effective methods are needed to evaluate PICC insertion and maintenance in patients undergoing long-term treatments. Although PICCs are not decisive factors affecting treatment outcomes, they are critical components that support the overall treatment process. However, there is a gap in economic evaluations related to these essential devices, leading to a lack of knowledge regarding their integration into the care pathway.

The primary aim of this study was to evaluate a health economic model that compares a new, potentially superior PICC design—ABGA PICCs—with the standard option over a 90-day follow-up period. This analysis aimed to fill existing knowledge gaps in economic evaluations and guide the integration of more effective PICC types into clinical practice, ultimately enhancing patient care and resource utilization.

## 2. Methods

### 2.1. Study Design and Compliance

This study adhered to the Consolidated Health Economic Evaluation Reporting Standards (CHEERS) to ensure the thoroughness and accuracy of reporting [11]. The observed objective was the cost-effectiveness of standard and AGBA PICCs, which incorporated potential catheter-related complications during the clinical trial into the models, including the failure of the first puncture, central line-associated bloodstream infections (CLABSIs), unknown fevers, thrombosis, and other local complications associated with catheter use.

**Figure 1:**
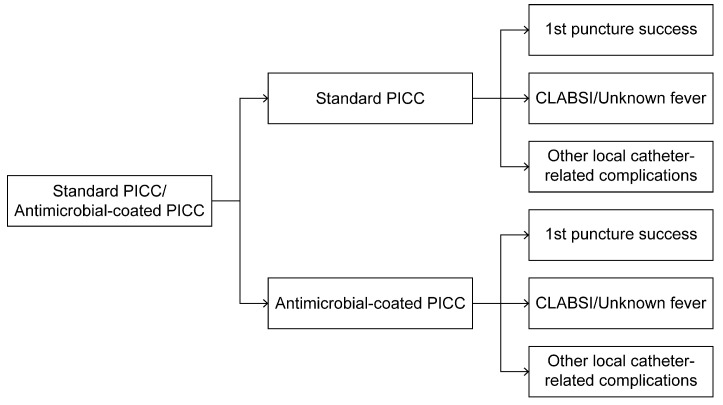
A decision tree framework for standard vs. antimicrobial-coated PICCs.

The target population comprised patients who were suspected of requiring a PICC; the considered time horizon, which refers to the common survival time of patients undergoing oncological treatment, was 5 years (Figure 1).

### 2.2. Target Population and Economic Perspective

Cost analyses were conducted from the perspective of a social reimbursement payer, reflecting the structure of China’s healthcare fund wherein the government bears most of the direct medical costs. This analysis is particularly pertinent given the rapid demographic shifts and escalating healthcare demands of an aging population. This study specifically targeted patients with cancer who were likely to require long-term PICC placement.

Cost analyses were conducted from a social reimbursement payer’s perspective, in line with an attempt to reduce the healthcare resources allocated to this treatment. In China’s reimbursement system, the government is the largest payer of direct costs, and the growth of the aging population is rapid; hence, the healthcare burden is substantial.

### 2.3. Conceptual Model: Decision Tree of Two PICCs Selected at >30 Days for Maintenance

Modeling is a tool for supporting decision making that anticipates and predicts the impacts of specific healthcare interventions on a group of patients or society [12,13,14]. A decision tree is a type of decision analysis model—used for economic evaluation in this study—that can be used to depict potential complications when a patient needs to undergo a PICC insertion procedure. The decision tree is mainly valued for its simplicity and transparency in describing options of interest, costs, and health outcomes indicated on a specific path or branch.

In the case of the two types of related complications, a hypothetical assumption was that all complications could be detected using laboratory tests. In this model, there were two alternative outcomes after prescribing a PICC: two branches indicating first-time-attempt insertion success or failure. When the patient accepted PICC insertion, the PICC indwelled in the patient’s body for approximately 15 days to 3 months, and catheter-related complications appeared in the models as branches. The possibilities for branches were fever, local complications, and complication-free status.

Catheter-related complications and booming prevention protocols have been sufficiently discussed since the late 1990s. For these protocol, there are three alternative outcomes: recovery from complications without catheter removal, recovery from complications with catheter removal, and the worsening of the situation [15,16,17,18]. If the catheter is removed, a new catheter needs to be inserted, and symptom-related treatments must be considered. The model examined in this study, thus, included some additional sections wherein these patients required multiple laboratory tests to confirm the diagnosis during the follow-up period.

### 2.4. Data Collection: Probabilities, Costs, and Quality-Adjusted Life Years (QALYs)

The model included the probabilities of PICC patient maintenance periods of >30 days at each node in the tree and branch. In this model, most of the data were collected based on two randomized clinical trials involving PICCs conducted in China. QALY data were collected based on the published literature, which was not directly observed through the two Chinese PICC randomized clinical trials [19,20]. Cost-per-procedure data for PICC insertion and maintenance from 2020 to 2023 were obtained from the official list of the Beijing Municipal Medical Insurance Bureau. All costs in the trial were reported in Renminbi (RMB); in this study, they were recalculated in US dollars using the official average currency conversion rate from the China Central Bank from January 2020 to December 2023 (RMB 678.03 = USD 100) [21,22]. Costs were categorized into laboratory tests, in-hospital care expenses, follow-up, catheter maintenance, catheter expenses, catheter-related complication treatments, and other treatments. The end of each branch accounted for several cost categories corresponding to all the steps conducted in specific pathways. The laboratory tests included blood tests, ultrasound tests, and radiography. In-hospital care and follow-up expenses were based on clinical routines. According to the protocol, all catheter-related complications required laboratory testing to confirm clinical inference, and the related treatment costs were obtained from real study data. Other treatment costs were based on real records and were difficult to predict based on model assumptions. For the catheter-related cost component, 95% bias-corrected confidence intervals were calculated using the Monte Carlo method with 10,000 random samples with replacement. The Monte Carlo method was used to model the probabilities of different outcomes, which were difficult to predict owing to the intervention of random variables, and helped to optimize the process parameters.

A literature review was conducted to gather the QALY data before and after transplantation for patients who underwent marrow transplantation [19,21]. The PubMed, Scopus, and Cochrane databases were searched. The timeframe was set to 10 years (2014–2023), and the search terms used were [(QALYs) OR (Health-Related Quality of Life)] AND (marrow transplant).

We processed data using the decision tree model and the Markov model, as provided by TreeAge Pro Healthcare (version 2023 R1.2), a software package specifically designed for health outcomes research from TreeAge Software, Inc., Williamstown, MA, USA).

### 2.5. ICER and Threshold

An incremental cost-effectiveness ratio (ICER) was calculated to determine whether AGBA PICCs were a cost-effective solution for patients with hematologic disease undergoing long-term intravenous infusion therapy. Because PICCs are devices, the ratios reflect the different costs of the two models and correspond to the same QALYs. The calculated ICER was compared with a threshold value that represents society’s willingness to pay (WTP) for better health outcomes per year, i.e., one QALY. The Chinese Minister of Health has no official guidelines regarding WTP; thus, we referred to the NICE guidelines. Related articles recommended that 1.76 times the gross domestic product be considered the threshold for non-life-saving technology; in this study, a threshold of RMB 113,120 (USD 16,884) was used [23,24,25,26,27].

### 2.6. Sensitivity Analyses

Sensitivity analyses were conducted based on the assumption that standard and AGBA PICCs influence the cost-effectiveness of the entire infusion treatment period [28,29]. Probabilistic sensitivity analyses were performed to estimate the ICER distribution. A cost-effectiveness plane was built, and the Monte Carlo model was constructed 10,000 times. Based on theoretical grounds, the net monetary benefit and WTP sensitivity analyses test the assumptions related to their respective levels in the catheter indwelling period using descriptive statistics for cost-per-patient data and varying other parameters [30].

## 3. Results

According to the clinical study data, the decision tree model’s simulated results showed that the mean healthcare cost per patient for standard PICCs is RMB 21,987.32 (USD 3242.82, at an average exchange rate of RMB 678.03 = USD 100), affecting 0.68 QALYs in 90 days. The mean healthcare cost per patient for AGBA PICCs is RMB 19,696.23 (USD 2904.92), affecting 0.73 QALYs in 90 days, thus resulting in incremental costs of RMB 2291.10 (USD 428.44). Because the catheter works as a channel, it does not impact QALYs; however, after the model’s simulation, standard PICCs gain −0.05 QALYs. The ICER for AGBA compared to standard PICCs is consistently centered at RMB 4271.31 (USD 629.96), with no variability across simulations.

### 3.1. Sensitivity Analysis

**Figure 2:**
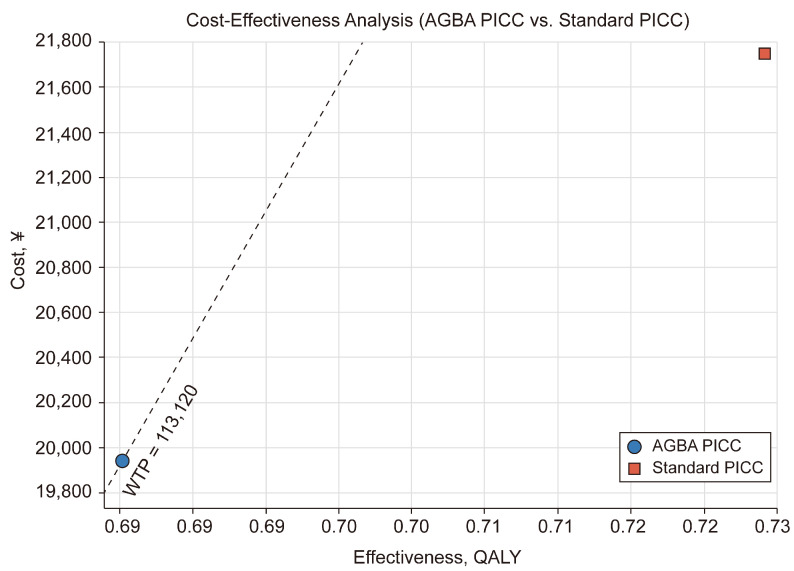
A cost-effectiveness analysis produced using TreeAge Pro 2024. WTP, willingness to pay; QALY, quality-adjusted life year.

A WTP threshold of RMB 113,120 (USD 16,683.63) per QALY is used to assess the cost-effectiveness of the two interventions. This cost-effectiveness analysis evaluates the economic and clinical value of AGBA compared to standard PICCs by examining the cost and effectiveness measured in QALYs (Figure 2). AGBA PICCs lie below the WTP threshold line, indicating their cost-effectiveness. In contrast, standard PICCs are positioned above the WTP line, reflecting higher costs and lower cost-effectiveness. This suggests that AGBA PICCs offer a better balance between costs and health outcomes, reinforcing their economic advantages.

**Figure 3:**
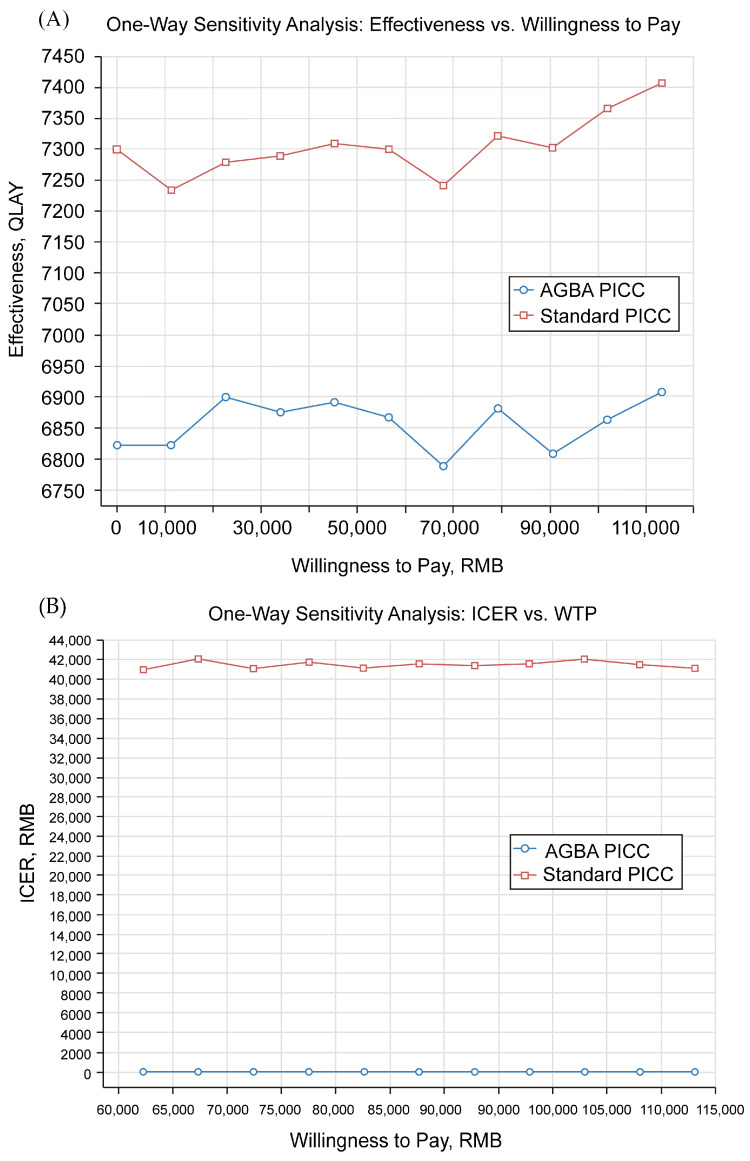
One-way sensitivity analysis derived from the following: (**A**) analysis of QALYs and willingness to pay for the two types of PICCs; (**B**) analysis of the ICER and willingness to pay. QALY, quality-adjusted life year; ICER, incremental cost-effectiveness ratio.

According to a one-way sensitivity analysis, a relationship between WTP and effectiveness (QALY) was observed for both AGBA and standard PICCs. The results indicated that standard PICCs consistently demonstrated higher cost per patient per QALY than AGBA PICCs across varying WTP thresholds (Figure 3). However, the difference in effectiveness was relatively stable, suggesting that the cost-effectiveness ratio may favor AGBA PICCs.

Another one-way sensitivity analysis of the relationship between the ICER and WTP for AGBA compared with standard PICCs was plotted against varying WTP thresholds (Figure 3). The ICER values fluctuated between RMB 41,000 (USD 6046.93) and RMB 42,000 (USD 6194.42) across different WTP levels, reflecting the variability in cost-effectiveness under different economic assumptions. The dashed vertical line at RMB 113,120 (USD 16,683.63) represents the threshold at which an intervention is considered cost-effective based on the predetermined WTP limit.

### 3.2. Monte Carlo Probabilistic Analysis

**Figure 4:**
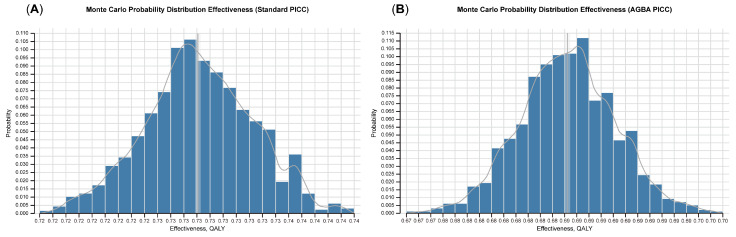
Monte Carlo probability distributions of effectiveness, measured in quality-adjusted life years (QALYs), for (**A**) AGBA PICCs and (**B**) standard PICCs.

**Figure 5:**
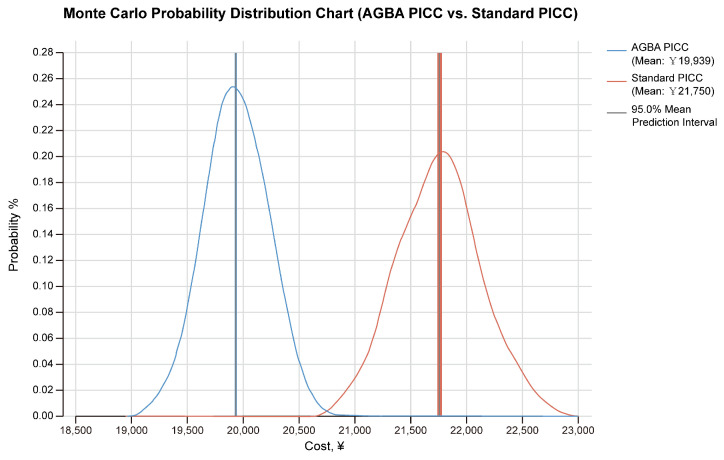
Monte Carlo probability distribution of costs for AGBA PICCs and standard PICCs.

A Monte Carlo probability distribution simulation was conducted for AGBA and standard PICCs to predict the QALY distribution. The results showed that the values of AGBA PICCs mostly clustered around 0.69 QALYs (Figure 4). Standard PICCs demonstrated a higher mean QALY value (~0.73) than AGBA PICCs. The distribution reflects the probabilistic nature of the effectiveness and highlights the variability in patient outcomes. The narrow 95% prediction interval around the AGBA PICC’s cost further reflects lower variability and greater cost predictability. In contrast, the standard PICC distribution exhibited a higher mean cost and slightly wider variability. The AGBA PICC curve’s relatively narrow spread indicates consistent effectiveness and cost. The distribution of the standard PICC is broader, indicating more variability and a higher risk of negative patient outcomes (Figure 5). 

## 4. Discussion

According to this health economic model, AGBA PICCs are a more cost-effective tool than standard PICCs; however, standard PICCs have marginally higher QALYs than AGBA PICCs. In this case, the ICER for AGBA PICCs compared to standard PICCs was RMB 4271.31 per QALY, which is far lower than the RMB 113,120 used as an informal WTP threshold in China. As the ICER values consistently remained below this threshold, AGBA PICCs were deemed cost-effective across the entire range of WTP values. This result reinforces the economic viability of AGBA PICCs, demonstrating that even under the most conservative estimates, the intervention remains within acceptable cost-effectiveness limits.

In the sensitivity analysis, AGBA PICCs proved to be a cost-effective option; however, there were some uncertainties regarding WTP and cost variations. This finding further supports the adoption of AGBA PICCs in clinical practice, especially in resource-constrained environments where cost containment is a priority.

While standard PICCs may yield marginally better health outcomes, the associated costs exceed the WTP threshold, limiting their economic feasibility. The cost savings and lower risks associated with AGBA PICCs provide a compelling case for their adoption in long-term treatment strategies. A potential obstacle to adoption may be the limited awareness of the chlorhexidine-coated products, as well as the absence of local evidence to support their efficacy.

These findings suggest that integrating AGBA PICCs into routine practice can optimize healthcare resource allocation without jeopardizing patient outcomes. This analysis serves as a valuable tool for policymakers and healthcare administrators seeking to enhance cost-efficiency while maintaining high standards of care. This model supports decision makers in optimizing resource allocation, emphasizing the importance of balancing economic constraints with clinical effectiveness.

## 5. Conclusions

In conclusion, AGBA PICCs are cost-effective alternatives that align with value-based care principles and provide a sustainable option for long-term treatment.

## Data Availability

All data are provided within the manuscript.

## Abbreviations

The following abbreviations are used in this manuscript:

AGBA	Chlorhexidine-coated PICC
CLABSI	Central-line-associated bloodstream infection
ICER	Incremental cost-effectiveness ratio
PICC	Peripherally inserted central catheter
QALY	Quality-adjusted life years
WTP	Willingness to pay
